# Immunological Interfaces: The COVID-19 Pandemic and Depression

**DOI:** 10.3389/fneur.2021.657004

**Published:** 2021-04-23

**Authors:** Austin Perlmutter

**Affiliations:** Independent Researcher, Portland, OR, United States

**Keywords:** depression, immunity, cytokine storm, cytokines, microglia, COVID-19, SARS- CoV-2, pandemic

## Abstract

Since the start of the spread of the coronavirus disease 2019 (COVID-19) pandemic, an international effort has sought to better characterize associated extra-pulmonary health sequelae. The acute and or chronic detrimental impact of SARS-CoV-2 infection on mental health, especially depression, is increasingly described. Simultaneously the pandemic has influenced depressive symptomatology by modifying economic, social and political structures, in addition to affecting daily routines. In both cases, associated immunological perturbations favoring a pro-inflammatory state could underlie an increased risk for depressive symptomatology. A resultant elevation in global depressive burden could further tax mental health care infrastructure and contribute to a range of worse health outcomes including diminished quality of life. This suggests a critical and time-sensitive need to better understand immune interfaces between depression and COVID-19.

## Introduction

The unprecedented spread of SARS-CoV-2 has created a global emphasis on the immune system and its role in COVID-19 disease risk, outcomes and therapeutics (1). Yet the importance of immunity in human health has increasingly expanded beyond infectious diseases. Alterations in immunological activation are now recognized for their role in diverse disease states (2–4). Cytological patterns of innate and adaptive immunity can indicate severity of disease burden and risk for complications in infectious and non-infectious conditions (5, 6). In the field of mental health, various immune cell and cytokine subsets are implicated in the pathogenesis of schizophrenia, anxiety and depression (7, 8). Among these data, an immunologic milieu characterized by elevated systemic inflammation has been repeatedly associated with the development of depressive symptomatology (9).

Prior to SARS-CoV-2, depression was already a worldwide epidemic with considerable negative impact on morbidity and mortality. It is estimated that globally 350 million people are affected by depression, and it is a leading cause of disability (10, 11). In addition to lowered quality of life, depression may also contribute to a shortened lifespan (12). Over the last century, a variety of hypotheses have explored biological underpinnings of depression and potential opportunities for treatment and prevention. Alterations in immunological pathways, especially increased low-grade systemic inflammation, are now the subject of extensive academic research (13). In a subset of patients with depression, elevated systemic inflammation is proposed to play a substantial role in disease pathogenesis (14).

**Figure 1:**
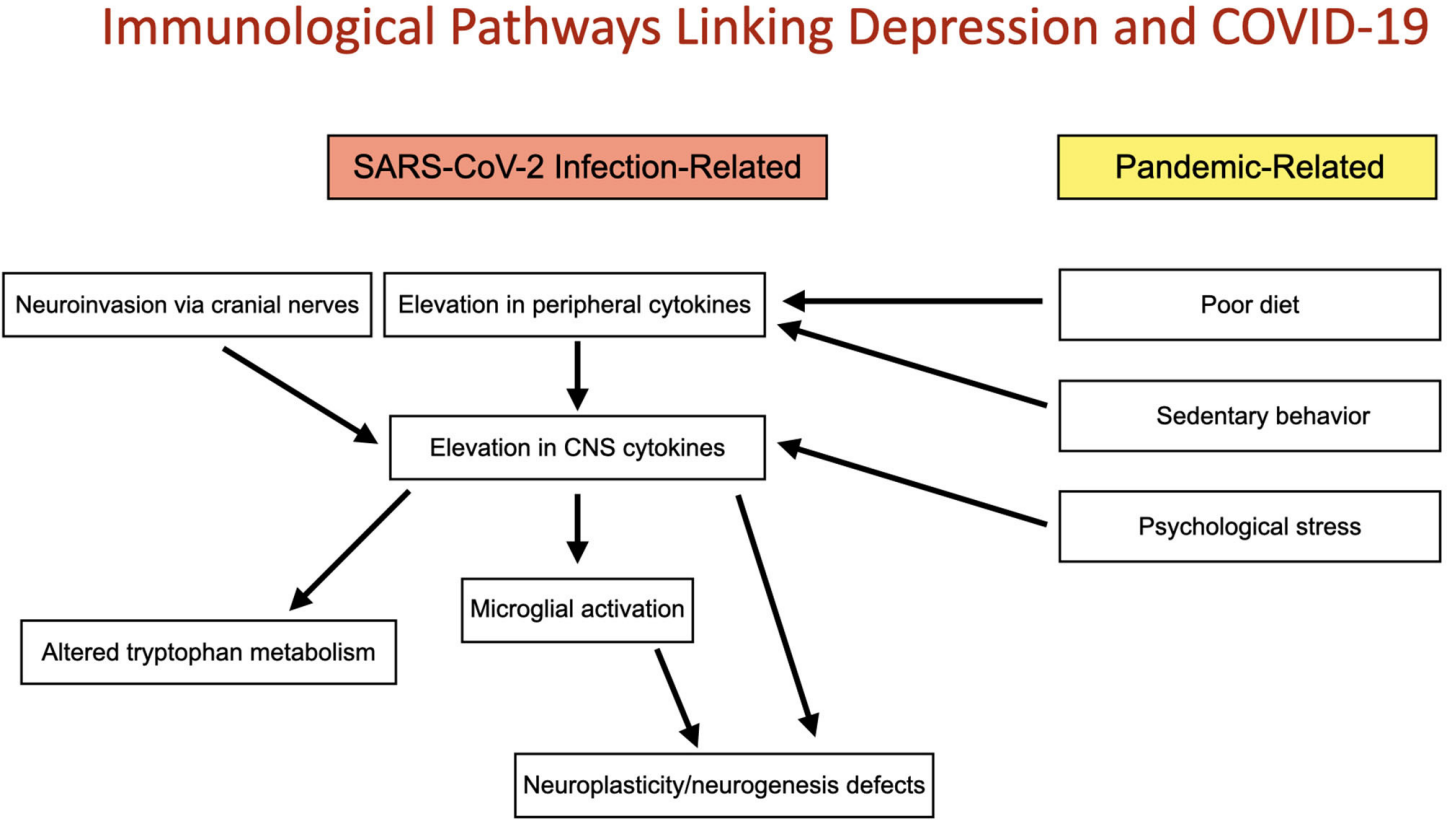
Immunological pathways linking depression and COVID-19: Two convergent pathways connect the COVID-19 pandemic with altered immune function and depression. These include direct immunological implications of SARS-CoV-2 infection and indirect, non-infectious pandemic-related changes in immune function induced by poor diet, sedentary behavior and psychological stress. Both pathways may act to increase risk for depression by elevation of CNS cytokines and subsequently microglial activation, altered tryptophan metabolism and deficits in neuroplasticity and neurogenesis.

COVID-19 has the potential to induce widespread immunological effects as a direct result of infection and indirectly, independent of infection by modifying behavior and thought patterns. This may preferentially shift the immunological milieu toward an inflammatory state and predispose to higher rates of depressive symptomatology. The present review focuses on immune pathways linking COVID-19 infection with risk for depression as well as putative non-infectious immune mechanisms by which SARS-CoV-2 could increase depressive burden (Figure 1).

## Review of Recent Literature

As the data around short and long-term consequences of the COVID-19 pandemic accumulate, evidence suggests an urgent need to focus on neuropsychiatric sequelae. Though over 1,800 publications jointly mention COVID-19 and depression, fewer than 100 discuss the terms “immunity,” “depression” and “COVID-19.” Themes in these articles include: the role of complementary therapies, the beneficial role of physical activity, and pharmacological consideration and bidirectional interactions between COVID-19 and depression with a focus on stress, cytokine storm, and long-term depressive outcomes related to SARS-CoV-2 infection.

The extant research includes mention of several complementary therapies purported to target shared immunological pathways in depression and COVID-19. These include the use of Ayurveda (15) traditional Chinese medicine (TCM) (16), and dietary interventions of curcumin (17) and omega-3 fatty acids (18). The use of selective serotonin reuptake inhibitors (SSRIs) (19) and oxytocin (20) in COVID-19 have also been considered for their immunomodulatory and mood-related properties, and the importance of exercise as an immunomodulator and potential anti-depressive has also been described (21).

Two recent reviews have focused specifically on molecular mechanisms linking immunity, COVID-19 and depression. In the first (22)) the authors emphasize parallel pathways of kynurenine (KYN) pathway activation by COVID-19 cytokine storm and angiotensin-converting enzyme 2 (ACE2) receptor effects in increased risk for depression. In the second (23), the authors emphasize the role of cytokine storm in potential psychological outcomes from COVID-19. Related molecular pathways are also briefly discussed in a recent cohort analysis (24).

## Existing Links Between Depression and SARS-COV-2

Worldwide, millions of cases of the infectious disease SARS-CoV-2 have been reported (25), accompanied by a near universal exposure to political, social and economic ripple effects. Early in the course of the pandemic, the immunological effects of the virus on human physiology were characterized by respiratory symptoms including severe pneumonia (26). In the coming months, extra-pulmonary manifestations of the virus were better described. These included cardiovascular, metabolic, hematologic, neurologic and dermatologic pathologies (27). Additional research highlighted a tax on mental health as a potential consequence of acute infection (28).

Academic and public focus has also expanded to the long-term effect of SARS-CoV-2 infection on human health (29). This has been called “post-acute COVID-19” or “long COVID” (30). Those who experience persistent symptoms for weeks or months after acute infection number in the thousands, and have created Facebook self-help groups, adopting the terminology “Long-Haul COVID” to describe their ongoing battle with health issues including worse cognition, low exercise tolerance, sleep problems, autonomic dysfunction as well as worsened mental health and autoimmunities (31–33). A recent study (34) more explicitly linked depressive psychopathology 3 months after hospitalization for COVID-19 pneumonia with elevated baseline scores on an index of immune activation and inflammation.

**Figure 2:**
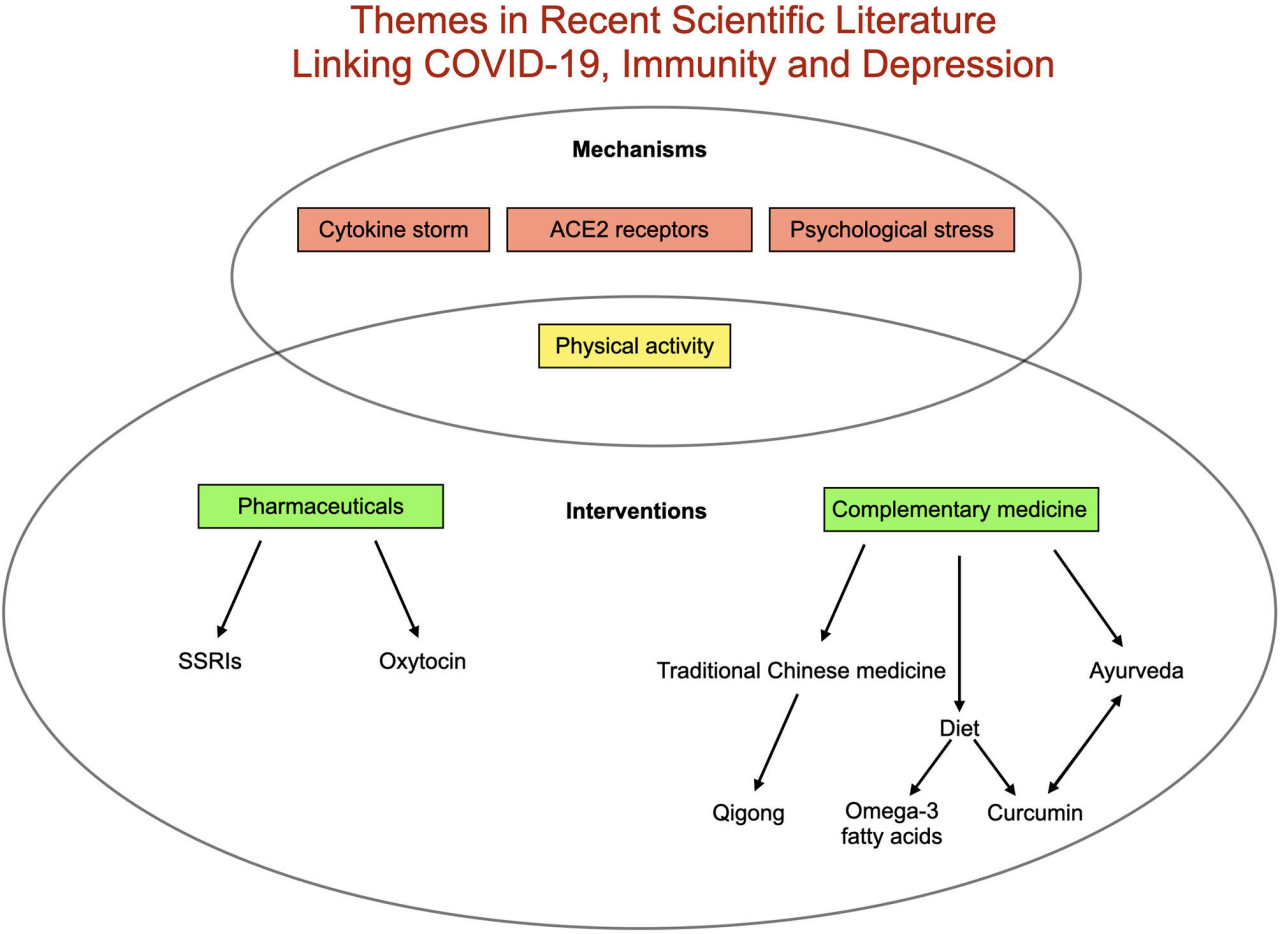
Summary of existing literature relating COVID-19, depression and immunity. Proposed mechanisms triggering depression related to COVID-19 include elevated inflammation (best represented in cytokine storm), pathways related to angiotensin-converting enzyme 2 (ACE2) receptors, decreased physical activity and increased psychological stress. Proposed interventions include a range of complementary therapies as well as pharmaceutical [(SSRIs (selective serotonin reuptake inhibitors) and oxytocin] in addition to increasing physical activity.

Finally, attention has increasingly turned to the indirect ramifications of the virus. In globally disrupting routines, the economy, access to care and social dynamics, the pandemic could alter health outcomes for billions. Early data suggest these impacts may prove especially relevant for mental health. Health care workers managing COVID-19 patients in China reported increased psychological strain, including higher rates of depressive symptoms (35). A population-based analysis of depressive symptoms in the US found a 3-fold increase in symptoms during the COVID-19 pandemic compared to before (36). In a recent survey of 130 countries, the World Health Organization (37) reported widespread disruption of mental health service for vulnerable populations (25). Patients with preexisting mental health and physical health conditions may be at particular risk for pandemic-related depression, and low social support and socioeconomic position may also confer increased risk (38). Additionally depression may itself confer heightened risk for further immune-mediated depressive symptoms through increased vulnerability to inflammatory immune activation after psychosocial stress (39). A summary of recently published themes linking COVID-19, immunity and depression can be found in Figure 2.

## Immune Dysfunction and Depression

Early evidence that inflammation could precipitate depressive symptoms was derived from hepatitis patients receiving immunotherapy with interferon alpha. Many of these patients developed psychiatric symptoms including symptoms of depression (40). Subsequent research showed that administration of low-dose endotoxin (e.g., lipopolysaccharide (LPS)) increases systemic markers of inflammation including tumor necrosis factor alpha (TNFα) and interleukin 6 (IL-6) and symptoms of depression (41). Research additionally demonstrates correlation between elevated inflammatory cytokines and depressive symptoms (9), and increased levels of the inflammatory markers high-sensitivity C-reactive protein (hs-CRP) and IL-6 have been shown to be risk factors for subsequent depression (42, 43).

Brain alterations are central to the current understanding of depression pathophysiology (44). However, the brain is generally regarded as having immune privilege, which limits its exposure to peripheral immune states including inflammation (45). It is now understood that there are three methods by which peripheral inflammation may reach and influence the brain (46). These include cytokine passage through the blood brain barrier (BBB), cytokine activation of peripheral afferent nerve fibers returning to the central nervous system (CNS) and the trafficking of immune cells into the brain. In addition, CNS immune function can be directly activated when antigens enter thorough cranial nerves. For example, pathogens may reach the CNS by way of the olfactory and trigeminal nerves (45), and vagal afferent fibers convey immune-modulating signals from gut bacteria to the brain (46).

## Neurological Immune Response in SARS-COV-2 Infection

In investigating the SARS-CoV-2 virus, research efforts have focused on the role of pre-existing immunological states as well as immune changes subsequent to infection. Direct correlations between COVID-19 outcomes and individual cytokines and immune cell populations indicate symptoms of COVID-19 are associated with elevations in interleukin 1 beta (IL-1β), IL-6, interleukin 10 (IL-10), and TNFα), as well as a general lymphopenia (47–49). Increased IL-6 may especially correlate with severity of COVID-19 (50).

An infection-related surge in proinflammatory mediators in COVID-19 has been called the cytokine storm or cytokine release syndrome. While consensus on the exact definition of the cytokine storm is debated, it is characterized by elevation in a range of immunological markers including interleukins, interferon-γ, TNF, chemokines and plasma proteins including complement and C-reactive protein (CRP) (51). In cytokine storm, hyperactivation of the inflammatory immune response may lead to significant collateral damage including respiratory distress, renal failure, liver injury and cardiomyopathy as well as neuropsychiatric issues (51). Up to 40% of people with COVID-19 have been reported to experience significant central nervous system (CNS) symptoms (52).

At this time, the precise methods by which the SARS-CoV-2 virus influences the CNS remain unclear. However, the widely cited symptoms of anosmia and dysgeusia suggest a high prevalence of CNS involvement (53), potentially via retrograde olfactory nerve transport (54). Research in prior coronaviruses has demonstrated coronavirus RNA in the human brain, suggesting a degree of neuroinvasion despite the virus's label as a respiratory pathogen (54). Peripheral immunological activation as a result of COVID-19 could also reach the CNS by transport through or disruption of the BBB (55, 56).

On entering the CNS, peripherally generated inflammatory mediators may amplify their effects on the brain by acting on microglial cells. Microglial activation represents a transition from a state of relative quiescence to a “primed” state in which the microglia increase production of cytokines and other inflammatory mediators (57). Microglia are implicated in both acute and chronic neurological complications of COVID-19 infection (58), and increased microglial activation has been demonstrated in post-mortem neuropathological analysis of brain samples from COVID-19 patients (59, 60).

Those with existing neuroimmunological diseases may have heightened vulnerability to depressive symptomatology as a result of the COVID-19 pandemic. For example, people with multiple sclerosis demonstrated elevated rates of psychological distress including depression after easing of lockdown measures (61). Parkinson's disease has been strongly correlated with neuroimmune alterations including increased neuroinflammation (62), and a recent survey demonstrated high rates of depressive symptomatology in this demographic (63). Additionally, patients with preexisting psychiatric diagnoses have been found to experience high rates of psychiatric symptoms including those related to depression in the context of COVID-19 lockdown measures, and immunological mechanisms have been proposed as a potential contributor (64).

Data demonstrating the long-term impact of COVID-19 on neuroimmune function remain limited. However, early survey results suggest that after acute infection, some experience residual symptoms of including fatigue, headache and anosmia, indicating a degree of persistent neurological alteration (65). Animal research implies a possibility for coronavirus-mediated neuronal damage as a result of alterations in glutamate homeostasis as well as potential for T cell-mediated demyeliation in susceptible hosts (66). SARS-CoV-2 infiltration of the olfactory bulb and subsequent polarization of microglial cells toward an inflammatory phenotype has also been proposed as a mechanism promoting neurodegenerative disease (67). As microglia mediate multiple neurological processes, chronic alterations in microglial populations as a result of COVID-19 could have significant impact on multiple health outcomes including depression. While much investigative focus has been on direct links between viral infection and neuroimmune alterations in otherwise healthy individuals, those with existing neuroimmunological conditions may be especially vulnerable to non-infectious psychological stressors stemming from the pandemic.

## Neuroplasticity and Neurogenesis

Neuroplasticity describes neural synaptic reorganization in response to environmental input. It is thought to form the basis for memory and learning (68). Impaired neuroplasticity is implicated in the pathogenesis of depression (69), a mechanism supported by alterations in brain functional connectivity (70) as well as loss of synapse-related genes and synapses in postmortem brain tissue of patients with depression (71).

Immune function is thought to exert a degree of control over neuroplasticity (72). This may occur in a dose-dependent manner with opposing effects at extremes of immune activation, as a low basal level of neuroinflammatory cytokines IL-1β and TNFα appears necessary for healthy neuroplasticity, with suppression at higher levels (73).

As regulators of CNS immunity, microglial cells are implicated in neuroplasticity. Microglia are thought to influence this process through glutamate homeostasis and production of inflammatory cytokines (74). On detection of homeostatic disturbance (e.g., metabolic, stress-related and pathogen-induced signals), microglia become activated, proliferating and producing inflammatory mediators (75). This may present a convergent mechanism by which peripheral immune activation and psychosocial stress could induce neuroinflammation, defects in neuroplasticity and eventually, depression.

Neurogenesis is the process of creating new neurons. Once thought restricted to early life, human research now shows that neurogenesis occurs in discrete zones of the brain into adulthood, including the hippocampus and lateral ventricle (76, 77). Like neuroplasticity, neurogenesis may underlie mechanisms of learning and memory (78). The neurogenesis hypothesis of depression proposes changes in the rate of neurogenesis in the subgranular zone of the dentate gyrus of the hippocampus in the pathophysiology of the disease (77).

Neuroplasticity and neurogenesis are affected by neurotrophic factors, compounds that bind to tyrosine kinase receptors and augment neuronal function, survival and development (79). Of these, much research has specifically focused on the role of brain-derived neurotrophic factor (BDNF) and its role in neuroplasticity, neurogenesis and depression (80, 81). Lowered levels of BDNF protein and BDNF gene expression are reported in both post-mortem brain tissue and in peripheral blood from depressed patients (82), and BDNF is increased by antidepressant therapies ranging from conventional antidepressants to electro-convulsive treatment (ECT) (83, 84).

Immunity plays a role in both BDNF expression and function. Administration of LPS reliably induces inflammation, including in the CNS (85). In animal models, LPS increases expression of inflammatory markers in the hippocampus and microglia (86) and decreases levels of BDNF (87). It is also notable that microglial cells regulate release of BDNF (88).

The aforementioned pathway suggests a molecular mechanism by which infection with COVID-19 could directly downregulate levels of BDNF. However, non-infectious effects of the pandemic may also play a role, as chronic stress is thought to have a deleterious effect on BDNF expression (89). This implies that healthy neuroplasticity and neurogenesis may be compromised as a result of infection and psychological stressors generated by COVID-19 pandemic.

## Serotonin and Tryptophan Metabolism

Decreased brain bioavailability of the tryptophan (TRP) metabolite serotonin (5-HT) underpins the psychopharmacology of the most commonly prescribed antidepressants (90). TRP metabolites including 5-HT engage in bidirectional interactions with the immune system.

5-HT may directly influence immune homeostasis by suppressing Th17 differentiation, increasing expression of T regulatory cells (Tregs) and promoting M2-polarization of macrophages (91). These immunological changes favor decreased inflammation and may speak to a role for serotonin in mediating inflammation-associated depressive symptoms.

More robust research focuses on the effects of immunity on TRP metabolism, including 5-HT availability. Enzymatic action on the essential amino acid TRP determines whether it is converted in 5-HT or shunted into the kynurenine (KYN) pathway. In general, the majority of tryptophan enters the KYN pathway, creating downstream metabolites including KYN, kynurenic acid (KYNA) and quinolinic acid (QUIN) (92).

Initiating enzymes in the KYN pathway are tryptophan 2,3-dioxygenase (TDO) and indolamine-2,3-dioxygenase (IDO). Notably, IDO is highly expressed in immune cells (93) and compared to TDO it is far more responsive to immunological signals (94). In the context of elevated pro-inflammatory cytokines including IL-1β and TNFα, IDO converts TRP to KYN, and shunts available TRP away from 5-HT production (95). Conversely, anti-inflammatory cytokines including interleukin 4 (IL-4) and IL-10 deactivate the IDO enzyme (96, 97). Recently, it has been proposed that by inducing cytokine storm and downregulating ACE2, the SARS-CoV-2 virus may increase levels of KYN pathways metabolites in the brain, increasing risk for depression (22).

The relative increase in KYN pathway activation as a result of inflammatory immune activation has been proposed to contribute to depression through 5-HT depletion, though more recent focus has shifted to the differential neuroactive effects of KYN metabolites (96). For example, KYNA may exert anti-depressant effects through N-Methyl-D-aspartate (NMDA) antagonist mechanisms resembling those of ketamine, while the NMDA agonist QUIN may have pro-depressive effects (96). While individual human trials are variable, there is some support for a decrease in KYNA and an increase in QUIN levels in depression (98).

**Figure 3:**
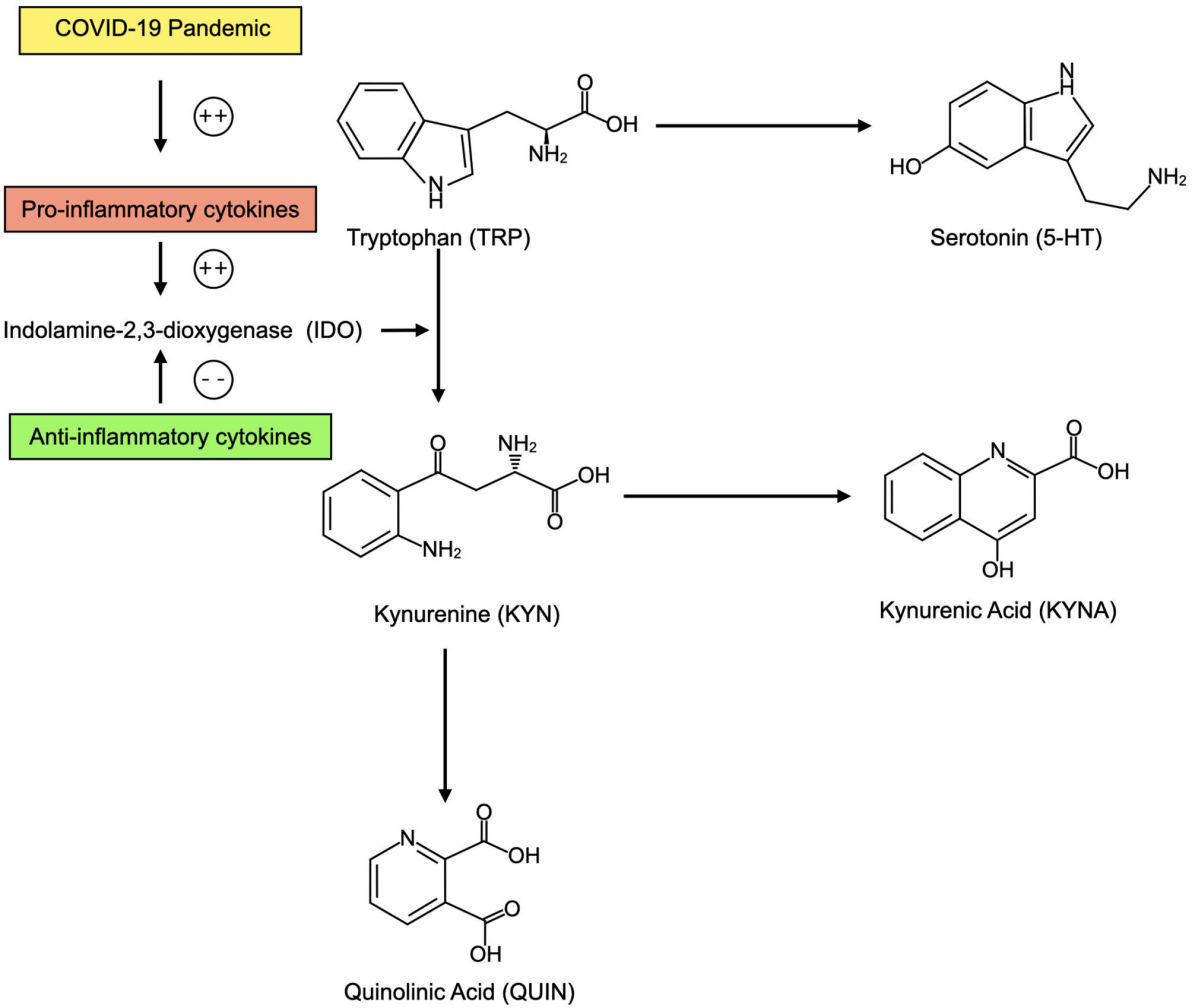
In the context of elevated inflammatory cytokines as a result of the COVID-19 pandemic, CNS tryptophan (TRP) may be preferentially converted into kynurenine (KYN) and its metabolites including kynurenic acid (KYNA) and quinolinic acid (QUIN). This may decrease availability of serotonin (5-HT). KYN metabolites may also contribute to depression. When anti-inflammatory cytokines are present, TRP may be preferentially converted into 5-HT.

In sum, an elevation in systemic inflammation as the direct result of infection with SARS-CoV-2 or as a result of pandemic-related behavioral changes and psychological stressors could predispose to relative brain 5-HT depletion and imbalance in KYN pathway metabolites that increase risk for depression (Figure 3).

## Psychological Stress

Increased psychological stress has been widely documented in response to COVID-19. Personal diagnosis of or the diagnosis of a close contact with COVID-19 have each been associated with elevated stress as well as with increased symptoms of depression (99). Political measures taken to reduce the spread of the virus have been associated with elevated stress internationally (100, 101). Exposure to content related to COVID-19 has also been linked to increased psychological stress. In a survey of American adults without prior history of a mental health condition, 15% reported 2 symptoms of psychological distress for at least 3 days in the past week, most commonly the sense of feeling nervous, anxious, or on edge (102). These symptoms were positively associated with social media and internet engagement with coronavirus content. Widespread food scarcity and economic instability as a result of the pandemic also pose a significant risk for elevated psychological stress (103).

Alterations in stress pathways including the hypothalamic-pituitary-adrenal (HPA) axis and sympathetic nervous system (SNS) are known risk factors for mental health conditions including anxiety and depression (104, 105). Early life exposure to major life stressors predicts a higher chance of developing depression in adulthood (106). At a cellular and molecular level, multiple stress-related alterations are seen in depression, including changes in levels of stress hormones, receptor expression of neurons and HPA responsiveness to glucocorticoids (107–109). These changes may exert their effects on mood by way of immune modulation in the CNS (110).

Elevated psychological stress is proposed to induce immunological alterations through a variety of dose and duration-dependent mechanisms. Acute as well as chronic psychosocial stressors are associated with elevated inflammation (111). Acute stress correlates with elevation in plasma IL-6, TNFα, IL-1β and IL-10, while chronic stress has been linked to low-grade inflammatory activation including elevations in CRP, IL-6 and TNFα (111, 112). It is particularly topical that caregiving stress correlates with elevations in IL-6, while teacher burnout has been correlated with systemic inflammation along a continuum of symptom severity (Rohelder, 2019) (113).

Compared with acute stressors, chronic stress may better predict the low-grade inflammatory immune activation correlated with depression (114). Proposed linking mechanisms include glucocorticoid receptor resistance and subsequent inability to down-regulate inflammatory pathways as well as stress-induced production of inflammatory cytokines from visceral fat (115). Visceral fat expresses high levels of stress-related adrenergic receptors and produces pro-inflammatory cytokines including TNFα and IL-6 (116). Chronic stress may additionally cause microglia to adopt a pro-inflammatory phenotype, contributing to neuroinflammation (117). Inflammation may in part be mediated through stress activation of the NLRP3 inflammasome (118). Taken together, multiple mechanisms link COVID-19 pandemic-related psychological stress with an inflammatory immune state that could promote depressive symptomatology.

## Physical Activity

COVID-19 related restrictions have sparked concern for decreased physical activity. In a recent survey of American adults, people active prior to COVID-19 restrictions reported a 32% reduction in physical activity (119). It is also notable that in this report, increased inactivity correlated with an increase in depressive symptoms. Similar results in American children suggest a decrease in physical activity and increase in sedentary activity since the spread of the pandemic within the United States (120).

Physical activity has been shown to be an effective therapy for unipolar depression, with a benefit comparable to antidepressants and psychotherapy (121, 122). Recently, existing data have been supplemented by Mendelian randomization techniques showing a potential causative role for the protective effect of physical activity in depression (123).

Physical activity is thought to positively affect multiple aspects of immune function, with a beneficial suppressive effect on inflammation (124). As exercise induces a transient elevation in inflammatory markers, these effects can appear paradoxical, and various explanations have been proposed. While acute exercise may rapidly increase inflammation, including levels of interleukin 1 (IL-1), TNFα and IL-6, these muscle-derived cytokines (myokines) may have local anti-inflammatory effect (125, 126). Though exercise increases IL-6 in the CNS, this is accompanied by a decrease in TNFα (127). It is suggested that IL-6 may therefore act as an anti-inflammatory cytokine in regions of the CNS by inhibiting TNFα, with an overall protective effect on neurons. Finally, exercise-induced elevation in inflammatory cytokines may be systemically balanced by a parallel increases in anti-inflammatory molecules, leading to a net anti-inflammatory effect (128).

## Diet

Early research during the COVID-19 pandemic suggests the potential for a negative impact on dietary patterns, potentially as a response to increased chronic stress and changes in habits (129, 130). For example, an increased consumption of “comfort foods” has been reported in response to COVID-19 (131). A survey of Italians found that unhealthy dietary choices were driven in part by a desire to alleviate poor mental health (132).

In both children and adults, data indicate a COVID-19-related increase in intake of prototypically inflammatory foods including refined carbohydrates and sugary drinks and in adults, a decreased intake of fish and fruit (133–135). This change suggests that in response to the pandemic, there may a trend toward a Western pattern diet, which predicts higher levels of inflammatory markers including CRP and IL-6 (136) as well as increased risk for depression (137).

Dietary influences on immune function are diverse. The role of macro and micronutrient deficiency in immune dysfunction is well-characterized, and dietary fiber, omega-3 fatty acids and polyphenols are also purported to play a role in healthier immune function (138). Conversely, preclinical data suggest a deficit in dietary fiber could promote lower levels of Treg cells (139). Increased dietary saturated fat may facilitate inflammation by activation of the toll-like receptor (TLR4) signaling pathway, while omega-3 fats have the opposing effect (140). Additionally, immunity may be affected by changes in the gut microbiome (141) as well as more acutely by a lipid, glucose or dietary LPS-mediated postprandial inflammatory response (142). In the context of the aforementioned, it is notable that interventional trials promoting dietary patterns rich in whole foods and low in refined carbohydrates, fast foods, sweetened drinks and processed meats have demonstrated efficacy in decreasing depressive symptoms as well as in lowering inflammatory burden (143–146).

As a whole, these data suggest that changes in dietary patterns may represent a behavioral variable in inflammatory modulation, and that psychological stress-mediated trends during COVID-19 could have a detrimental effect on mood by increasing preference for less healthful foods. Emphasis on the consumption of a less processed diet and increased access to more nutrient-rich foods could represent a potential offset to diet-related immunological effects on depressive symptoms as a result of the pandemic.

## Discussion

Depression and COVID-19 demonstrate shared patterns of immunological function, especially around a pro-inflammatory state characterized by elevation in cytokines including IL-6, TNFα, and IL-1β. SARS-CoV-2 may increase immunological risk for depression through direct infection-related influences on the CNS, or through associated behavioral shifts in diet, physical activity and psychological stress which subsequently promote an inflammatory immune state. Insight into the immunological intersections between depression and SARS-CoV-2 may help in the creation of strategies to mitigate of depression risk during the COVID-19 pandemic.

## Author Contributions

The author confirms being the sole contributor of this work and has approved it for publication.

## Conflict of Interest

AP receives consulting fees from Big Bold Health.

## References

[1] Stephens DS McElrath MJ. COVID-19 and the Path to Immunity. JAMA. (2020) 324:1279–81. 10.1001/jama.2020.16656 32915201 PMC12177933

[2] Trott DW Harrison DG. The immune system in hypertension. Adv Physiol Educ. (2014) 38:20–4. 10.1152/advan.00063.2013 24585465 PMC4459918

[3] Maeda K Caldez MJ Akira S. Innate immunity in allergy. Allergy. (2019) 74:1660–74. 10.1111/all.13788 30891811 PMC6790574

[4] Betjes MG. Immune cell dysfunction and inflammation in end-stage renal disease. Nat Rev Nephrol. (2013) 9:255–65. 10.1038/nrneph.2013.44 23507826

[5] Thomas MR Lip GY. Novel risk markers and risk assessments for cardiovascular disease. Circ Res. (2017) 120:133–49. 10.1161/CIRCRESAHA.116.309955 28057790

[6] Huang H Xu R Lin F Bao C Wang S Ji C. High circulating CD39+ regulatory T cells predict poor survival for sepsis patients. Int J Infect Dis. (2015) 30:57–63. 10.1016/j.ijid.2014.11.006 25461658

[7] Drexhage RC Weigelt K van Beveren N Cohen D Versnel MA Nolen WA. Immune and neuroimmune alterations in mood disorders and schizophrenia. Int Rev Neurobiol. (2011) 101:169–201. 10.1016/B978-0-12-387718-5.00007-9 22050852

[8] Salim S Chugh G Asghar M. Inflammation in anxiety. Adv Protein Chem Struct Biol. (2012) 88:1–25. 10.1016/B978-0-12-398314-5.00001-5 22814704

[9] Osimo EF Pillinger T Rodriguez IM Khandaker GM Pariante CM Howes OD. Inflammatory markers in depression: a meta-analysis of mean differences and variability in 5,166 patients and 5,083 controls. Brain Behav Immun. (2020) 87:901–9. 10.1016/j.bbi.2020.02.010 32113908 PMC7327519

[10] Lim GY Tam WW Lu Y Ho CS Zhang MW Ho RC. Prevalence of depression in the community from 30 countries between 1994 and 2014. Sci Rep. (2018) 8:1–10. 10.1038/s41598-018-21243-x 29434331 PMC5809481

[11] Friedrich MJ. Depression is the leading cause of disability around the world. JAMA. (2017) 317:1517. 10.1001/jama.2017.3826 28418490

[12] Cuijpers P Smit F. Excess mortality in depression: a meta-analysis of community studies. J Affect Disord. (2002) 72:227–36. 10.1016/S0165-0327(01)00413-X 12450639

[13] Miller AH Raison CL. The role of inflammation in depression: from evolutionary imperative to modern treatment target. Nat Rev Immunol. (2016) 16:22. 10.1038/nri.2015.5 26711676 PMC5542678

[14] Raison CL Miller AH. Role of inflammation in depression: implications for phenomenology, pathophysiology and treatment. Inflamm Psychiatry. (2013) 28:33–48. 10.1159/000343966 25224889

[15] Rajkumar RP. Ayurveda and COVID-19: where psychoneuroimmunology and the meaning response meet. Brain Behav Immun. (2020) 87:8–9. 10.1016/j.bbi.2020.04.056 32334064 PMC7175849

[16] Ma K Wang X Feng S Xushan X Zhang H Rahaman A. From the perspective of Traditional Chinese Medicine: treatment of mental disorders in COVID-19 survivors. Biomed Pharmacother. (2020) 132:110810. 10.1016/j.biopha.2020.110810 33053508 PMC7524684

[17] Soni VK Mehta A Shukla D Kumar S Vishvakarma NK. Fight COVID-19 depression with immunity booster: Curcumin for psychoneuroimmunomodulation. Asian J Psychiatry. (2020) 53:102378. 10.1016/j.ajp.2020.102378 32916441 PMC7462590

[18] Chang JPC Pariante CM Su KP. Omega-3 fatty acids in the psychological and physiological resilience against COVID-19. Prostaglandins Leukot Essent Fatty Acids. (2020) 161:102177. 10.1016/j.plefa.2020.102177 33031994 PMC7516470

[19] Hamed MGM Hagag RS. The possible immunoregulatory and anti-inflammatory effects of selective serotonin reuptake inhibitors in coronavirus disease patients. Med Hypotheses. (2020) 144:110140. 10.1016/j.mehy.2020.110140 32768893 PMC7382922

[20] Thakur P Shrivastava R Shrivastava VK. Oxytocin as a Potential Adjuvant against COVID-19 Infection. Endocrine Metab Immune Disord Drug Targets. (2020). 10.2174/1871530320666200910114259. [Epub ahead of print]. 32914732

[21] Burtscher J Burtscher M Millet GP. (Indoor) isolation, stress and physical inactivity: vicious circles accelerated by Covid-19?. Scand J Med Sci Sports. (2020) 30:1544–5. 10.1111/sms.13706 32374894 PMC7267366

[22] Bouças AP Rheinheimer J Lagopoulos J. Why Severe COVID-19 patients are at greater risk of developing depression: a molecular perspective. Neuroscientist, (2020):1073858420967892. 10.1177/1073858420967892. [Epub ahead of print]. 33135582

[23] Debnath M Berk M Maes M. Changing dynamics of psychoneuroimmunology during the COVID-19 pandemic. Brain Behav Immun Health. (2020) 5:100096. 10.1016/j.bbih.2020.100096 32566934 PMC7295528

[24] Mazza MG De Lorenzo R Conte C Poletti S Vai B Bollettini B. COVID-19 Bio B, Outpatient Clinic Study group. Anxiety and depression in COVID-19 survivors: Role of inflammatory and clinical predictors. Brain Behav Immun. (2020) 89:594–600. 10.1016/j.bbi.2020.07.037 32738287 PMC7390748

[25] World Health Organization. COVID-19 Weekly Epidemiological Update. (2020). Available online at: https://www.who.int/publications/m/item/weekly-epidemiological-update-12-january-2021 (accessed January 18, 2020).

[26] Huang C Wang Y Li X Ren L Zhao J Hu Y. Clinical features of patients infected with 2019 novel coronavirus in Wuhan, China. Lancet. (2020) 395:497–506. 10.1016/S0140-6736(20)30183-5 31986264 PMC7159299

[27] Gupta A Madhavan MV Sehgal K Nair N Mahajan S Sehrawat T. Extrapulmonary manifestations of COVID-19. Nat Med. (2020) 26:1017–32. 10.1038/s41591-020-0968-3 32651579 PMC11972613

[28] Rogers JP Chesney E Oliver D Pollak TA McGuire P Fusar-Poli P. Psychiatric and neuropsychiatric presentations associated with severe coronavirus infections: a systematic review and meta-analysis with comparison to the COVID-19 pandemic. Lancet Psychiatry. (2020) 7:611–27. 10.1016/S2215-0366(20)30203-0 32437679 PMC7234781

[29] Carfì A Bernabei R Landi F. Persistent symptoms in patients after acute COVID-19. JAMA. (2020) 324:603–5. 10.1001/jama.2020.12603 32644129 PMC7349096

[30] Greenhalgh T Knight M Buxton M Husain L. Management of post-acute covid-19 in primary care. BMJ. (2020) 370:m3026. 10.1136/bmj.m3026 32784198

[31] Perego E Callard F Stras L Melville-Jóhannesson B Pope R Alwan NA. Why the patient-made term'long covid'is needed. Wellcome Open Res. (2020) 5:224. 10.12688/wellcomeopenres.16307.1

[32] Nath A. Long-haul COVID. Neurology. (2020) 95:559–60. 10.1212/WNL.0000000000010640 32788251

[33] Rodríguez Y Novelli L Rojas M De Santis M Acosta-Ampudia Y Monsalve D. Autoinflammatory and autoimmune conditions at the crossroad of COVID-19. J Autoimmun. (2020) 114:102506. 10.1016/j.jaut.2020.102506 32563547 PMC7296326

[34] Gennaro MM Mariagrazia P De Lorenzo R Cristiano M Sara P Roberto F. Persistent psychopathology and neurocognitive impairment in COVID-19 survivors: effect of inflammatory biomarkers at three-month follow-up. Brain Behav Immun. (2021). 10.1016/j.bbi.2021.02.021. [Epub ahead of print]. 33639239 PMC7903920

[35] Lai J Ma S Wang Y Cai Z Hu J Wei N. Factors associated with mental health outcomes among health care workers exposed to coronavirus disease 2019. JAMA Netw Open. (2020) 3:e203976. 10.1001/jamanetworkopen.2020.3976 32202646 PMC7090843

[36] Ettman CK Abdalla SM Cohen GH Sampson L Vivier PM Galea S. Prevalence of depression symptoms in US adults before and during the COVID-19 pandemic. JAMA Netw Open. (2020) 3:e2019686. 10.1001/jamanetworkopen.2020.19686 32876685 PMC7489837

[37] World Health Organization. COVID-19 Disrupting Mental Health Services in Most Countries. Available online at: https://www.who.int/news/item/05-10-2020-covid-19-disrupting-mental-health-services-in-most-countries-who-survey (accessed January 5, 2021).

[38] Iob E Frank P Steptoe A Fancourt D. Levels of severity of depressive symptoms among at-risk groups in the UK during the COVID-19 pandemic. JAMA Netw Open. (2020) 3:e2026064. 10.1001/jamanetworkopen.2020.26064 33104209 PMC7588938

[39] Fagundes CP Glaser R Hwang BS Malarkey WB Kiecolt-Glaser JK. Depressive symptoms enhance stress-induced inflammatory responses. Brain Behav Immun. (2013) 31:172–6. 10.1016/j.bbi.2012.05.006 22634107 PMC3518610

[40] Renault PF Hoofnagle JH Park Y Mullen KD Peters M Jones D. Psychiatric complications of long-term interferon alfa therapy. Arch Intern Med. (1987) 147:1577–80. 10.1001/archinte.1987.00370090055011 3307672

[41] Kotulla S Elsenbruch S Roderigo T Brinkhoff A Wegner A Engler H. Does human experimental endotoxemia impact negative cognitions related to the self? Front Behav Neurosci. (2018) 12:183. 10.3389/fnbeh.2018.00183 30186124 PMC6113574

[42] Pasco JA Nicholson GC Williams LJ Jacka FN Henry MJ Kotowicz M. Association of high-sensitivity C-reactive protein with de novo major depression. Br J Psychiatry. (2010) 197:372–7. 10.1192/bjp.bp.109.076430 21037214

[43] Gimeno D Kivimäki M Brunner EJ Elovainio M De Vogli R Steptoe A. Associations of C-reactive protein and interleukin-6 with cognitive symptoms of depression: 12-year follow-up of the Whitehall II study. Psychol Med. (2009) 39:413. 10.1017/S0033291708003723 18533059 PMC2788760

[44] Li BJ Friston K Mody M Wang HN Lu HB Hu DW. A brain network model for depression: From symptom understanding to disease intervention. CNS Neurosci Ther. (2018) 24:1004–19. 10.1111/cns.12998 29931740 PMC6490158

[45] Forrester JV McMenamin PG Dando SJ. CNS infection and immune privilege. Nat Rev Neurosci. (2018) 19:655–71. 10.1038/s41583-018-0070-8 30310148

[46] Bonaz B Bazin T Pellissier S. The vagus nerve at the interface of the microbiota-gut-brain axis. Front Neurosci. (2018) 12:49. 10.3389/fnins.2018.00049 29467611 PMC5808284

[47] Pedersen SF Ho YC. SARS-CoV-2: a storm is raging. J Clin Invest. (2020) 130:2202–5. 10.1172/JCI137647 32217834 PMC7190904

[48] Lin L Zhong C Rao S Lin H Huang R Chen F. Clinical characteristics of 78 cases of patients infected with coronavirus disease 2019 in Wuhan, China. Exp Ther Med. (2020) 21:1–1. 10.3892/etm.2020.9439 33235616 PMC7678631

[49] Liu B Li M Zhou Z Guan X Xiang Y. Can we use interleukin-6 (IL-6) blockade for coronavirus disease 2019 (COVID-19)-induced cytokine release syndrome (CRS)?. J Autoimmun. (2020) 111:102452. 10.1016/j.jaut.2020.102452 32291137 PMC7151347

[50] Zhu Z Cai T Fan L Lou K Hua X Huang Z. Clinical value of immune-inflammatory parameters to assess the severity of coronavirus disease 2019. Int J Infect Dis. (2020) 95:332–9. 10.1016/j.ijid.2020.04.041 32334118 PMC7195003

[51] Fajgenbaum DC June CH. Cytokine Storm. N Engl J Med. (2020) 383:2255–73. 10.1056/NEJMra2026131 33264547 PMC7727315

[52] Murta V Villarreal A Ramos AJ. Severe acute respiratory syndrome coronavirus 2 impact on the central nervous system: are astrocytes and microglia main players or merely bystanders?. ASN Neuro. (2020) 12:1759091420954960. 10.1177/1759091420954960 32878468 PMC7476346

[53] Lozada-Nur F Chainani-Wu N Fortuna G Sroussi H. Dysgeusia in COVID-19: possible mechanisms and implications. Oral Surg Oral Med Oral Pathol Oral Radiol. (2020) 130:344. 10.1016/j.oooo.2020.06.016 32703719 PMC7320705

[54] Arbour N Day R Newcombe J Talbot PJ. Neuroinvasion by human respiratory coronaviruses. J Virol. (2000) 74:8913–21. 10.1128/JVI.74.19.8913-8921.2000 10982334 PMC102086

[55] Banks WA Kastin AJ Broadwell RD. Passage of cytokines across the blood-brain barrier. Neuroimmunomodulation. (1995) 2:241–8. 10.1159/000097202 8963753

[56] Varatharaj A Galea I. The blood-brain barrier in systemic inflammation. Brain Behav Immun. (2017) 60:1–12. 10.1016/j.bbi.2016.03.010 26995317

[57] Li JW Zong Y Cao XP Tan L Tan L. Microglial priming in Alzheimer's disease. Ann Transl Med. (2018) 6:176. 10.21037/atm.2018.04.22 29951498 PMC5994530

[58] Tremblay ME Madore C Bordeleau M Tian L Verkhratsky A. Neuropathobiology of COVID-19: the role for glia. Front Cell Neurosci. (2020) 14:592214. 10.3389/fncel.2020.592214 33304243 PMC7693550

[59] Matschke J Lütgehetmann M Hagel C Sperhake JP Schröder AS Edler C. Neuropathology of patients with COVID-19 in Germany: a post-mortem case series. Lancet Neurol. (2020) 19:919–29. 10.1016/S1474-4422(20)30308-2 33031735 PMC7535629

[60] Hanley B Naresh KN Roufosse C Nicholson AG Weir J Cooke GS. Histopathological findings and viral tropism in UK patients with severe fatal COVID-19: a post-mortem study. Lancet Microbe. (2020) 1:e245–53. 10.1016/S2666-5247(20)30115-4 32844161 PMC7440861

[61] Zanghì A D'Amico E Luca M Ciaorella M Basile L Patti F. Mental health status of relapsing-remitting multiple sclerosis Italian patients returning to work soon after the easing of lockdown during COVID-19 pandemic: a monocentric experience. Mult Scler Relat Disord. (2020) 46:102561. 10.1016/j.msard.2020.102561 33045494 PMC7532774

[62] Troncoso-Escudero P Parra A Nassif M Vidal RL. Outside in: unraveling the role of neuroinflammation in the progression of parkinson's disease. Front Neurol. (2018) 9:860. 10.3389/fneur.2018.00860 30459700 PMC6232883

[63] Feeney MP Xu Y Surface M Shah H Vanegas-Arroyave N Chan AK. The impact of COVID-19 and social distancing on people with Parkinson's disease: a survey study. npj Parkinsons Dis. (2021) 7:1–10. 10.1038/s41531-020-00153-8 33479241 PMC7820020

[64] Hao F Tan W Jiang L Zhang L Zhao X Zou Y. Do psychiatric patients experience more psychiatric symptoms during COVID-19 pandemic and lockdown? A case-control study with service and research implications for immunopsychiatry. Brain Behav Immun. (2020) 87:100–6. 10.1016/j.bbi.2020.04.069 32353518 PMC7184991

[65] Sudre CH Murray B Varsavsky T Graham MS Penfold RS Bowyer RC. Attributes and predictors of Long-COVID: analysis of COVID cases and their symptoms collected by the Covid Symptoms Study App. medRxiv. (2020). 10.1101/2020.10.19.20214494

[66] Desforges M Le Coupanec A Dubeau P Bourgouin A Lajoie L Dubé M. Human coronaviruses and other respiratory viruses: underestimated opportunistic pathogens of the central nervous system?. Viruses. (2020) 12:14. 10.3390/v12010014 31861926 PMC7020001

[67] Mahalaxmi I Kaavya J Mohana Devi S Balachandar V. COVID-19 and olfactory dysfunction: a possible associative approach towards neurodegenerative diseases. J Cell Physiol. (2020) 236:763–70. 10.1002/jcp.29937 32697344 PMC7405062

[68] Albert PR. Adult neuroplasticity: a new “cure” for major depression?. JJPN. (2019) 44:147. 10.1503/jpn.190072 31038297 PMC6488487

[69] Price RB Duman R. Neuroplasticity in cognitive and psychological mechanisms of depression: an integrative model. Mol Psychiatry. (2020) 25:530–43. 10.1038/s41380-019-0615-x 31801966 PMC7047599

[70] Duman RS Aghajanian GK Sanacora G Krystal JH. Synaptic plasticity and depression: new insights from stress and rapid-acting antidepressants. Nat Med. (2016) 22:238–49. 10.1038/nm.4050 26937618 PMC5405628

[71] Kang HJ Voleti B Hajszan T Rajkowska G Stockmeier CA Licznerski P. Decreased expression of synapse-related genes and loss of synapses in major depressive disorder. Nat Med. (2012) 18:1413–7. 10.1038/nm.2886 22885997 PMC3491115

[72] Hayley S. The neuroimmune-neuroplasticity interface and brain pathology. Front Cell Neurosci. (2014) 8:419. 10.3389/fncel.2014.00419 25538568 PMC4255592

[73] Rizzo FR Musella A De Vito F Fresegna D Bullitta S Vanni V. Tumor necrosis factor and interleukin-1β modulate synaptic plasticity during neuroinflammation. Neural Plast. (2018) 2018:8430123. 10.1155/2018/8430123 29861718 PMC5976900

[74] Khairova RA Machado-Vieira R Du J Manji HK. A potential role for pro-inflammatory cytokines in regulating synaptic plasticity in major depressive disorder. Int J Neuropsychopharmacol. (2009) 12:561–78. 10.1017/S1461145709009924 19224657 PMC2771334

[75] Singhal G Baune BT. Microglia: an interface between the loss of neuroplasticity and depression. Front Cell Neurosci. (2017) 11:270. 10.3389/fncel.2017.00270 28943841 PMC5596091

[76] Eriksson PS Perfilieva E Björk-Eriksson T Alborn AM Nordborg C Peterson DA. Neurogenesis in the adult human hippocampus. Nat Med. (1998) 4:1313–7. 10.1038/3305 9809557

[77] Hanson ND Owens MJ Nemeroff CB. Depression, antidepressants, and neurogenesis: a critical reappraisal. Neuropsychopharmacology. (2011) 36:2589–602. 10.1038/npp.2011.220 21937982 PMC3230505

[78] Kitabatake Y Sailor KA Ming GL Song H. Adult neurogenesis and hippocampal memory function: new cells, more plasticity, new memories?. Neurosurg Clin. (2007) 18:105–13. 10.1016/j.nec.2006.10.008 17244558 PMC5439504

[79] Huang EJ Reichardt LF. Neurotrophins: roles in neuronal development and function. Annu Rev Neurosci. (2001) 24:677–736. 10.1146/annurev.neuro.24.1.677 11520916 PMC2758233

[80] Habtemariam S. The brain-derived neurotrophic factor in neuronal plasticity and neuroregeneration: new pharmacological concepts for old and new drugs. Neural Regen Res. (2018) 13:983. 10.4103/1673-5374.233438 29926822 PMC6022464

[81] Yang T Nie Z Shu H Kuang Y Chen X Cheng J. The Role of BDNF on Neural Plasticity in Depression. Front Cell Neurosci. (2020) 14:82. 10.3389/fncel.2020.00082 32351365 PMC7174655

[82] Cattaneo A Cattane N Begni V Pariante CM Riva MA. The human BDNF gene: peripheral gene expression and protein levels as biomarkers for psychiatric disorders. Transl Psychiatry. (2016) 6:e958. 10.1038/tp.2016.214 27874848 PMC5314126

[83] Sen S Duman R Sanacora G. Serum brain-derived neurotrophic factor, depression, and antidepressant medications: meta-analyses and implications. Biol Psychiatry. (2008) 64:527–32. 10.1016/j.biopsych.2008.05.005 18571629 PMC2597158

[84] Polyakova M Schroeter ML Elzinga BM Holiga S Schoenknecht P De Kloet ER. Brain-derived neurotrophic factor and antidepressive effect of electroconvulsive therapy: systematic review and meta-analyses of the preclinical and clinical literature. PLoS ONE. (2015) 10:e0141564. 10.1371/journal.pone.0141564 26529101 PMC4631320

[85] Brooks D Barr LC Wiscombe S McAuley DF Simpson AJ Rostron AJ. Human lipopolysaccharide models provide mechanistic and therapeutic insights into systemic and pulmonary inflammation. Eur Respir J. (2020) 56:1901298. 10.1183/13993003.01298-2019 32299854

[86] Golia MT Poggini S Alboni S Garofalo S Albanese NC Viglione A. Interplay between inflammation and neural plasticity: both immune activation and suppression impair LTP and BDNF expression. Brain Behav Immun. (2019) 81:484–94. 10.1016/j.bbi.2019.07.003 31279682

[87] Guan Z Fang J. Peripheral immune activation by lipopolysaccharide decreases neurotrophins in the cortex and hippocampus in rats. Brain Behav Immun. (2006) 20:64–71. 10.1016/j.bbi.2005.04.005 15922558

[88] Ferrini F De Koninck. Y. Microglia control neuronal network excitability via BDNF signalling. Neural Plast. (2013) 2013:429815. 10.1155/2013/429815 24089642 PMC3780625

[89] Duman RS Monteggia LM. A neurotrophic model for stress-related mood disorders. Biol Psychiatry. (2006) 59:1116–27. 10.1016/j.biopsych.2006.02.013 16631126

[90] Holsboer F. The corticosteroid receptor hypothesis of depression. Neuropsychopharmacology. (2000) 23:477–501. 10.1016/S0893-133X(00)00159-7 11027914

[91] Wan M Ding L Wang D Han J Gao P. Serotonin: a potent immune cell modulator in autoimmune diseases. Front Immunol. (2020) 11:186. 10.3389/fimmu.2020.00186 32117308 PMC7026253

[92] Badawy AA Guillemin G. The Plasma [Kynurenine]/[Tryptophan] ratio and indoleamine 2, 3-dioxygenase: time for appraisal. Int J Tryptophan Res. (2019) 12:1178646919868978. 10.1177/1178646919868978 31488951 PMC6710706

[93] Moffett JR Arun P Puthillathu N Vengilote R Ives JA Badawy AA. Quinolinate as a Marker for kynurenine metabolite formation and the unresolved question of NAD+ synthesis during inflammation and infection. Front Immunol. (2020) 11:31. 10.3389/fimmu.2020.00031 32153556 PMC7047773

[94] Yeung AW Terentis AC King NJ Thomas SR. Role of indoleamine 2, 3-dioxygenase in health and disease. Clin Sci. (2015) 129:601–72. 10.1042/CS20140392 26186743

[95] Myint AM Kim YK. Cytokine–serotonin interaction through IDO: a neurodegeneration hypothesis of depression. Med Hypotheses. (2003) 61:519–25. 10.1016/S0306-9877(03)00207-X 14592780

[96] Jeon SW Kim YK. Inflammation-induced depression: Its pathophysiology and therapeutic implications. J Neuroimmunol. (2017) 313:92–8. 10.1016/j.jneuroim.2017.10.016 29153615

[97] Weiss G Murr C Zoller H Haun M Widner B Ludescher C. Modulation of neopterin formation and tryptophan degradation by Th1-and Th2-derived cytokines in human monocytic cells. Clin Exp Immunol. (1999) 116:435. 10.1046/j.1365-2249.1999.00910.x 10361231 PMC1905306

[98] Ogyu K Kubo K Noda Y Iwata Y Tsugawa S Omura Y. Kynurenine pathway in depression: a systematic review and meta-analysis. Neurosci Biobehav Rev. (2018) 90:16–25. 10.1016/j.neubiorev.2018.03.023 29608993

[99] Gallagher MW Zvolensky MJ Long LJ Rogers AH Garey L. The impact of Covid-19 experiences and associated stress on anxiety, depression, and functional impairment in American adults. Cognit Ther Res. (2020) 44:1043–51. 10.1007/s10608-020-10143-y 32904454 PMC7456202

[100] Odriozola-González P Planchuelo-Gómez Á Irurtia MJ de Luis-García R. Psychological symptoms of the outbreak of the COVID-19 confinement in Spain. J Health Psychol. [Preprint]. (2020). 10.31234/osf.io/mq4fg 33124471

[101] Rossi R Socci V Talevi D Mensi S Niolu C Pacitti F. COVID-19 pandemic and lockdown measures impact on mental health among the general population in Italy. Front Psychiatry. (2020) 11:790. 10.3389/fpsyt.2020.00790 32848952 PMC7426501

[102] Holingue C Badillo-Goicoechea E Riehm KE Veldhuis CB Thrul J Johnson R. Mental distress during the COVID-19 pandemic among US adults without a pre-existing mental health condition: Findings from American trend panel survey. Prev Med. (2020) 139:106231. 10.1016/j.ypmed.2020.106231 32758507 PMC7846292

[103] Klassen S Murphy S. Equity as both a means and an end: lessons for resilient food systems from COVID-19. World Dev. (2020) 136:105104. 10.1016/j.worlddev.2020.105104 32834385 PMC7402645

[104] Juruena MF Eror F Cleare AJ Young AH. The role of early life stress in HPA axis and anxiety. Anxiety Disord. (2020) 1191:141–53. 10.1007/978-981-32-9705-0_9 32002927

[105] Tofoli SMDC Baes CVW Martins CMS Juruena M. Early life stress, HPA axis, and depression. Psychol Neurosci. (2011) 4:229–34. 10.3922/j.psns.2011.2.00833318074

[106] Cohen S Janicki-Deverts D Miller GE. Psychological stress and disease. JAMA. (2007) 298:1685–7. 10.1001/jama.298.14.1685 17925521

[107] Nemeroff CB Widerlov E Bissette G Walleus H Karlsson I Eklund K. Elevated concentrations of CSF corticotropin-releasing factor-like immunoreactivity in depressed patients. Science. (1984) 226:1342–4. 10.1126/science.6334362 6334362

[108] Raadsheer FC Hoogendijk WJ Stam FC Tilders FJ Swaab DF. Increased numbers of corticotropin-releasing hormone expressing neurons in the hypothalamic paraventricular nucleus of depressed patients. Neuroendocrinology. (1994) 60:436–44. 10.1159/000126778 7824085

[109] Heuser I Yassouridis A Holsboer F. The combined dexamethasone/CRH test: a refined laboratory test for psychiatric disorders. J Psychiatr Res. (1994) 28:341–56. 10.1016/0022-3956(94)90017-5 7877114

[110] Cruz-Pereira JS Rea K Nolan YM O'Leary OF Dinan TG Cryan JF. Depression's unholy trinity: dysregulated stress, immunity, the microbiome. Annu Rev Psychol. (2020) 71:49–78. 10.1146/annurev-psych-122216-011613 31567042

[111] Rohleder N. Stress and inflammation–The need to address the gap in the transition between acute and chronic stress effects. Psychoneuroendocrinology. (2019) 105:164–71. 10.1016/j.psyneuen.2019.02.021 30826163

[112] Marsland AL Walsh C Lockwood K John-Henderson NA. The effects of acute psychological stress on circulating and stimulated inflammatory markers: a systematic review and meta-analysis. Brain Behav Immun. (2017) 64:208–19. 10.1016/j.bbi.2017.01.011 28089638 PMC5553449

[113] von Känel R Bellingrath S Kudielka BM. Association between burnout and circulating levels of pro-and anti-inflammatory cytokines in schoolteachers. J Psychosom Res. (2008) 65:51–9. 10.1016/j.jpsychores.2008.02.007 18582612

[114] Maydych V. The interplay between stress, inflammation, and emotional attention: relevance for depression. Front Neurosci. (2019) 13:384. 10.3389/fnins.2019.00384 31068783 PMC6491771

[115] Cohen S Janicki-Deverts D Doyle WJ Miller GE Frank E Rabin BS. Chronic stress, glucocorticoid receptor resistance, inflammation, disease risk. Proc Natl Acad Sci U S A. (2012) 109:5995–9. 10.1073/pnas.1118355109 22474371 PMC3341031

[116] Black PH. The inflammatory consequences of psychologic stress: relationship to insulin resistance, obesity, atherosclerosis and diabetes mellitus, type II. Med Hypotheses. (2006) 67:879–91. 10.1016/j.mehy.2006.04.008 16781084

[117] Rohan Walker F Nilsson M Jones K. Acute and chronic stress-induced disturbances of microglial plasticity, phenotype and function. Curr Drug Targets. (2013) 14:1262–76. 10.2174/13894501113149990208 24020974 PMC3788324

[118] Weber MD Frank MG Tracey KJ Watkins LR Maier SF. Stress induces the danger-associated molecular pattern HMGB-1 in the hippocampus of male Sprague Dawley rats: a priming stimulus of microglia and the NLRP3 inflammasome. J Neurosci. (2015) 35:316–24. 10.1523/JNEUROSCI.3561-14.2015 25568124 PMC4287150

[119] Meyer J McDowell C Lansing J Brower C Smith L Tully M. Changes in physical activity and sedentary behavior in response to COVID-19 and their associations with mental health in 3052 US adults. Int J Environ Res Public Health. (2020) 17:6469. 10.3390/ijerph17186469 32899495 PMC7559240

[120] Dunton G Do B Wang S. Early Effects of the COVID-19 Pandemic on Physical Activity and Sedentary Behavior in US Children. BMC Public Health. (2020) 20:1351. 10.1186/s12889-020-09429-3 32887592 PMC7472405

[121] Kvam S Kleppe CL Nordhus IH Hovland A. Exercise as a treatment for depression: a meta-analysis. J Affect Disord. (2016) 202:67–86. 10.1016/j.jad.2016.03.063 27253219

[122] Morres ID Hatzigeorgiadis A Stathi A Comoutos N Arpin-Cribbie C Krommidas C. Aerobic exercise for adult patients with major depressive disorder in mental health services: a systematic review and meta-analysis. Depress Anxiety. (2019) 36:39–53. 10.1002/da.22842 30334597

[123] Choi KW Chen CY Stein MB Klimentidis YC Wang MJ Koenen KC. Assessment of bidirectional relationships between physical activity and depression among adults: a 2-sample mendelian randomization study. JAMA Psychiatry. (2019) 76:399–408. 10.1001/jamapsychiatry.2018.4175 30673066 PMC6450288

[124] Nieman DC Wentz LM. The compelling link between physical activity and the body's defense system. J Sport Health Sci. (2019) 8:201–17. 10.1016/j.jshs.2018.09.009 31193280 PMC6523821

[125] Ostrowski K Rohde T Asp S Schjerling P Pedersen BK. Pro-and anti-inflammatory cytokine balance in strenuous exercise in humans. J Physiol. (1999) 515:287–91. 10.1111/j.1469-7793.1999.287ad.x 9925898 PMC2269132

[126] Walsh NP Gleeson M Shephard RJ Gleeson M Woods JA Bishop N. Position statement part one: immune function and exercise. Exerc Immunol Rev. (2011) 17:6–63. 21446352

[127] Svensson M Lexell J Deierborg T. Effects of physical exercise on neuroinflammation, neuroplasticity, neurodegeneration, and behavior: what we can learn from animal models in clinical settings. Neurorehabil Neural Repair. (2015) 29:577–89. 10.1177/1545968314562108 25527485

[128] Gleeson M Bishop NC Stensel DJ Lindley MR Mastana SS Nimmo MA. The anti-inflammatory effects of exercise: mechanisms and implications for the prevention and treatment of disease. Nat Rev Immunol. (2011) 11:607–15. 10.1038/nri3041 21818123

[129] Mattioli AV Sciomer S Cocchi C Maffei S Gallina S. Quarantine during COVID-19 outbreak: changes in diet and physical activity increase the risk of cardiovascular disease. Nutr Metab Cardiovasc Dis. (2020) 30:1409–17. 10.1016/j.numecd.2020.05.020 32571612 PMC7260516

[130] Marty L de Lauzon-Guillain B Labesse M Nicklaus S. Food choice motives and the nutritional quality of diet during the COVID-19 lockdown in France. Appetite. (2020) 157:105005. 10.1016/j.appet.2020.105005 33068666 PMC7558232

[131] Scarmozzino F Visioli F. Covid-19 and the subsequent lockdown modified dietary habits of almost half the population in an Italian sample. Foods. (2020) 9:675. 10.3390/foods9050675 32466106 PMC7278864

[132] Di Renzo L Gualtieri P Cinelli G Bigioni G Soldati L Attinà A. Psychological aspects and eating habits during COVID-19 home confinement: Results of EHLC-COVID-19 Italian Online Survey. Nutrients. (2020) 12:2152. 10.3390/nu12072152 32707724 PMC7401000

[133] Pietrobelli A Pecoraro L Ferruzzi A Heo M Faith M Zoller T. Effects of COVID-19 lockdown on lifestyle behaviors in children with obesity living in Verona, Italy: a longitudinal study. Obesity. (2020) 28:1382–5. 10.1002/oby.22861 32352652 PMC7267384

[134] Deschasaux-Tanguy M Druesne-Pecollo N Esseddik Y de Edelenyi FS Alles B Andreeva VA. Diet and physical activity during the COVID-19 lockdown period (March-May 2020): results from the French NutriNet-Sante cohort study. Am J Clin Nutr. (2021) 113:924–38. 10.1101/2020.06.04.20121855 33675635 PMC7989637

[135] Pahwa R Jialal I. Chronic Inflammation. Treasure Island, FL: StatPearls Publishing, (2019). 29630225

[136] Lopez-Garcia E Schulze MB Fung TT Meigs JB Rifai N Manson JE. Major dietary patterns are related to plasma concentrations of markers of inflammation and endothelial dysfunction. Am J Clin Nutr. (2004) 80:1029–35. 10.1093/ajcn/80.4.1029 15447916

[137] Li Y Lv MR Wei YJ Sun L Zhang JX Zhang HG. Dietary patterns and depression risk: a meta-analysis. Psychiatry Res. (2017) 253:373–82. 10.1016/j.psychres.2017.04.020 28431261

[138] Barrea L Muscogiuri G Frias-Toral E Laudisio D Pugliese G Castellucci B. Nutrition and immune system: from the Mediterranean diet to dietary supplementary through the microbiota. Crit Rev Food Sci Nutr. (2020):1–25. 10.1080/10408398.2020.1792826. [Epub ahead of print]. 32691606

[139] Daïen CI Pinget GV Tan JK Macia L. Detrimental impact of microbiota-accessible carbohydrate-deprived diet on gut and immune homeostasis: an overview. Front Immunol. (2017) 8:548. 10.3389/fimmu.2017.00548 28553291 PMC5427073

[140] Rogero MM Calder PC. Obesity, inflammation, toll-like receptor 4 and fatty acids. Nutrients. (2018) 10:432. 10.3390/nu10040432 29601492 PMC5946217

[141] Brown K DeCoffe D Molcan E Gibson DL. Diet-induced dysbiosis of the intestinal microbiota and the effects on immunity and disease. Nutrients. (2012) 4:1095–119. 10.3390/nu4081095 23016134 PMC3448089

[142] Calder PC Ahluwalia N Brouns F Buetler T Clement K Cunningham K. Dietary factors and low-grade inflammation in relation to overweight and obesity. Br J Nutr. (2011) 106:S1–78. 10.1017/S0007114511005460 22133051

[143] Jacka FN O'Neil A Opie R Itsiopoulos C Cotton S Mohebbi M. A randomised controlled trial of dietary improvement for adults with major depression (the ‘SMILES'trial). BMC Med. (2017) 15:1–13. 10.1186/s12916-017-0791-y 28137247 PMC5282719

[144] Parletta N Zarnowiecki D Cho J Wilson A Bogomolova S Villani A. A Mediterranean-style dietary intervention supplemented with fish oil improves diet quality and mental health in people with depression: A randomized controlled trial (HELFIMED). Nutr Neurosci. (2019) 22:474–87. 10.1080/1028415X.2017.1411320 29215971

[145] Francis HM Stevenson RJ Chambers JR Gupta D Newey B Lim CK. A brief diet intervention can reduce symptoms of depression in young adults–A randomised controlled trial. PLoS ONE. (2019) 14:e0222768. 10.1371/journal.pone.0222768 31596866 PMC6784975

[146] Casas R Sacanella E Urpí-Sardà M Corella D Castaner O Lamuela-Raventos RM. Long-term immunomodulatory effects of a mediterranean diet in adults at high risk of cardiovascular disease in the PREvención con DIeta MEDiterránea (PREDIMED) randomized controlled trial. J Nutr. (2016) 146:1684–93. 10.3945/jn.115.229476 27440261

